# Exporting Socialist Health Care: Soviet Humanitarianism in the Global South

**DOI:** 10.1177/00220094251393617

**Published:** 2025-11-24

**Authors:** Siobhán Hearne

**Affiliations:** 5292 University of Manchester, UK

**Keywords:** USSR, humanitarianism, Global South, Soviet healthcare, medical aid, Cold War

## Abstract

This article focuses on three Soviet Red Cross overseas hospitals to examine the contradictions and challenges of socialist humanitarianism in the context of the Cold War. The development of socialist humanitarianism in the Soviet context was part of the broader foreign policy reorientation away from isolationism and towards robust internationalist engagement after the death of Iosif Stalin. Healthcare assistance was an integral component of this engagement and one in which the Soviet Red Cross played an active role through training local medical personnel, delivering healthcare assistance, and the management and maintenance of overseas hospitals. This article focuses on Soviet Red Cross hospitals in the cities of Tehran, Addis Ababa, and Phnom Penh. From the Soviet side, these hospitals were flagship projects of socialist humanitarianism and ‘gifts’ of high-quality, modern healthcare that symbolized the USSR's friendship with formerly colonized peoples and commitment to aiding the development of various states in the Global South. However, in practice, the hospitals’ functioning was shaped by the volatile political context, issues of budgeting and capitalization, and the clash between ideas about anticolonial solidarity and Soviet civilisational superiority.

‘Asia. Africa. The Hindustan peninsula. Their inhabitants are very familiar with the Soviet symbols of mercy and assistance: the Red Cross and the Red Crescent’, began an interview with Soviet doctors working overseas that was published in the magazine *Sovetskii Krasnyi Krest* (*The Soviet Red Cross*) in late 1970.^1^ All the interviewees were either current or former employees of overseas hospitals that were managed by the Soviet Red Cross and claimed that they had been motivated to embark upon medical work abroad by their commitment to the high ideals of socialist humanitarianism. ‘Selflessness and a sense of international duty is what has always guided us. We all went to Algeria at the behest of our hearts’, noted one doctor working at the Algerian-Soviet Friendship Hospital, which had been established in the town of Lakhdaria in 1966. However, rather than merely a matter of the heart, socialist humanitarianism was an important part of Soviet foreign policy; one that was characterized by a complex set of negotiations between the USSR's government and leaders in the Global South and enacted on-the-ground by Soviet medical professionals in often volatile political situations. This article uses Soviet Red Cross overseas hospitals as a lens for exploring the contradictions and challenges of Soviet humanitarianism during the Cold War.

Soviet overseas hospitals were part of a longer tradition of bilateral healthcare cooperation that stretched back to the 1920s and 1930s and accelerated following Stalin's death in 1953.^2^ The first Soviet Red Cross hospitals were constructed in the mid-1940s, mainly in areas where the Soviet Army had a presence, including North Korea, China, and Iran.^3^ After Stalin's death, Soviet medical cooperation became more widespread, and Soviet public health experts began to embed themselves more directly within global health networks.^4^ From the mid-1950s onwards, the Soviet government also began to meaningfully engage with leaders of newly independent states within the decolonizing world, driven by a mixture of anti-imperialist sentiments and a desire to convince national elites to adopt socialist development models.^5^ Under Khrushchev and later Brezhnev, the Soviet government signed bilateral agreements on development aid – understood as a mix of cultural exchange, military support, and economic and technical assistance – with the governments of numerous countries in the Global South. In contrast to Western models of development, Soviet visions prioritized the public sector and welfare systems over the establishment of commercial ventures, which usually meant supporting large-scale industrial and infrastructural projects that drew upon Soviet materials and expertise.^6^ Healthcare and medical assistance were also integral components of these interactions, but they have not yet been integrated into scholarship on Soviet development aid.

Through examining Soviet overseas hospitals, this article incorporates the USSR into the rich scholarship on socialist medical internationalism in the Global South.^7^ In delivering medications and equipment, training local medical personnel, and establishing hospitals and clinics, socialist states attempted to export their national healthcare models to numerous countries across decolonizing Africa and Asia.^8^ This bilateral medical internationalist engagement was framed as an act of solidarity with formerly colonized peoples and evidence of the socialist world's firm commitment to anticolonialism and national liberation. Unlike military or economic support, healthcare was regarded as a more neutral form of aid. This meant that national elites in non-aligned countries, and even those rather hostile the socialist bloc, could accept offers of support in this field, especially when aid was channelled through a humanitarian organization, rather than a government.

In the case of the USSR, the Soviet Red Cross (or as it was officially known, the Union of Red Cross and Red Crescent Societies of the USSR) was one of the most important channels for delivering medical aid and healthcare assistance to the Global South.^9^ The Soviet Red Cross was a parastatal organization with a visible presence both within the USSR and the international Red Cross movement. The organization worked closely with Soviet state ministries, and its work was financed by a mix of state funding and membership dues collected from its millions of members. The Central Committee of the Communist Party of the Soviet Union (CPSU) selected the organization's leadership and tightly controlled the scope of its overseas operations by allocating its foreign currency budget and retaining the power of veto over any international activities.^10^ The Soviet Red Cross also worked closely with the Geneva-based League of Red Cross Societies and was an enthusiastic participant in its Development Programme, which aimed to provide technical, financial, and material assistance to new Red Cross and Red Crescent societies in the Global South.^11^

Both domestically and internationally, the Soviet Red Cross presented a vision of humanitarianism that was split across Cold War divides, wherein socialist humanitarianism was superior to the capitalist version. As Severyan Dyakonov argues, the fact that the Soviet Red Cross ‘redefined humanitarianism as an inherently socialist value, distinct from Western philanthropy’ undergirded the organization's focus on providing long-term healthcare cooperation through the establishment of overseas medical facilities and by training local medical personnel.^12^ Soviet Red Cross leadership claimed that national Red Cross societies were more ‘philanthropic’ in the capitalist West and relied primarily on funding from private donors, whereas in socialist states the Red Cross had the strong support of state ministries.^13^ This framing was likely not only used to assert moral superiority over the numerous much wealthier Red Cross branches in the West, but also to justify state oversight of socialist national societies. For the Soviet Red Cross, the strong links between the Red Cross and the state was not a violation of the Red Cross fundamental principle of independence but instead reflected the moral and ideological superiority of socialist humanitarianism, as well as the Soviet government’s extraordinary dedication to pursuing universally shared humanitarian values.^14^

Despite the Soviet Red Cross's proclamations of the distinctiveness of socialist humanitarianism, certain aspects of its implementation in practice bared resemblance to its much-derided capitalist counterpart. Just like with other medical missions originating in the capitalist West or state socialist Europe, the relationship between Soviet medical workers and the local population was shaped by linguistic and practical difficulties, as well as imagined civilizational hierarchies that emphasized the ‘backwardness’ of postcolonial societies.^15^ There was also a gulf between the donor country's rhetorical commitments to providing healthcare assistance to countries in the Global South and the funding allocated to achieve this in practice. In the case of the USSR, this gulf pushed the Soviet Red Cross to adopt financial models that would have been otherwise ideologically unacceptable in order to ensure that overseas medical facilities could function. Issues with budgeting and capitalization meant that Soviet Red Cross hospitals predominantly functioned to showcase Soviet medicine, rather than provide universal healthcare to local populations. Therefore, Soviet humanitarianism was inflected by what historians have described as the ‘main distinctive feature of socialist globalization’: the intertwining of the issue of profitability with principles of solidarity and mutual assistance.^16^

This article focuses on three overseas hospitals that were fully or partly managed by the Soviet Red Cross in Iran, Ethiopia, and Cambodia. Taking inspiration from the pioneering scholarship of Bogdan C. Iacob and Iris Borowy, I approach these hospitals as emblematic of the power asymmetries and conflicting strategic and economic interests that underwrote East-South medical bilateralism.^17^ The Soviet side presented these hospitals as ‘gifts’ of high-quality, modern healthcare that symbolized the USSR's friendship with formerly colonized peoples and commitment to aiding the development of various states in the Global South.^18^ Tehran, Addis Ababa, and Phnom Penh were also sites of geopolitical competition in the fields of medicine and healthcare, which meant that each Soviet Red Cross hospital was just one of many medical institutions that were established and maintained by foreign powers. The article examines the functioning of these hospitals from the mid-1950s until the mid-1980s, a period bookended by the Soviet government's accelerating and declining interest in engaging with national elites in the Global South. While this period was marked by significant shifts in East-South relations, particularly in relation to trade and development, these broader trends did not have a significant impact upon the day-to-day functioning of the three Soviet Red Cross hospitals under consideration.^19^ Therefore, rather than proceeding chronologically, this article adopts a thematic approach to provide an overview of common issues that shaped Soviet humanitarianism across this broader period within three vastly different national contexts.

This article draws upon letters, reports, and publications produced by the leadership of the Soviet Red Cross, as well as correspondence between members of the organization's Executive Committee and Soviet medical personnel working in Tehran, Addis Ababa, and Phnom Penh. While this source base offers insight into the transnational history of Soviet humanitarianism, it is largely skewed towards the actions and reactions of a handful of powerful elites in Moscow. In the absence of sources from the Iranian, Ethiopian, and Cambodian sides, it is difficult to ascertain how the hospitals were received by the broader population. Nevertheless, Soviet sources on these hospitals illuminate how Soviet humanitarianism functioned in practice and provide crucial insight into what Paula Michaels and Stéphanie Panichelli-Batalla describe as ‘medical internationalism as a lived experience in the Cold War’.^20^ The article begins by contextualizing the establishment of the three hospitals within the history of the USSR's diplomatic relations with Iran, Ethiopia, and Cambodia. The narrative then moves on to explore how Soviet humanitarianism was shaped by the political and economic context of late socialism, as well as entanglements between ideas about anticolonial solidarity and Soviet civilizational superiority.

The Soviet Red Cross opened their hospital in Tehran in 1947, shortly after Stalin's attempts to retain Iranian territory after the end of the Second World War and secure a Soviet oil concession had resolutely failed, and at a time when Soviet-Iranian relations were rapidly deteriorating.^21^ It is highly likely that the establishment of the hospital was connected with the numerous military-scientific expeditions conducted by Soviet experts in the early 1940s during the Soviet-British occupation of Iran.^22^ In the late 1940s, the Iranian government pursued a series of anti-communist policies, including banning the Iranian communist party *Tudeh* and placing tight restrictions upon Soviet cultural programmes and organizations operating within the country.^23^ In 1953, the death of Stalin and the US- and British-instigated coup reconfigured the leadership of both the USSR and Iran, but Khrushchev and the Shah remained suspicious of one another for the majority of his premiership and exchange between the two countries was limited to the cultural sphere.^24^ Despite these difficulties, the Soviet Red Cross hospital in Tehran became a well-established medical institution with a wide range of specialisms, including surgery, urology, obstetrics and gynaecology, paediatrics, neurology, radiology, otolaryngology, dentistry, and dermato-venerology.^25^ The number of seconded Soviet specialists gradually increased from 25 in 1959 to 63 by the mid-1970s.^26^ By this point, the hospital had 130 inpatient beds and an outpatient clinic that could accommodate 400 appointments per day.^27^

The Soviet Red Cross also opened their hospital in Ethiopia in 1947. The Addis Ababa hospital was highly symbolic, not least because it fit within a longer trajectory of medical cooperation that stretched back to the late nineteenth century.^28^ Just like in Tehran, the Soviet Red Cross hospital in Addis Ababa opened against the backdrop of tense Ethiopian-Soviet relations. Throughout the late 1940s, Moscow's pro-independence stance towards former Italian-administered African colonies like Eritrea was not well received in Addis Ababa, and Emperor Haile Selassie signed a number of important agreements with the United States on security, economic, and technical assistance in the early 1950s.^29^ Towards the end of the decade, Khrushchev's rise to power and Haile Selassie's growing dissatisfaction with the US in light of the Suez Crisis and Washington's stance on Somaliland recalibrated Moscow-Addis Ababa relations. The Emperor took steps to diversify foreign aid and investments through establishing economic and cultural links with the socialist and non-aligned world.^30^ Haile Selassie visited the USSR in 1959 and signed a number of agreements with the Soviet government on economic and technical assistance and secured an enormous loan of 90 million rubles, the largest credit provided by Moscow to a Sub-Saharan African country.^31^ Just like the hospital in Tehran, the Soviet Red Cross hospital in Addis Ababa was a well-regarded medical institution with numerous specialisms (surgery, obstetrics, gynaecology, paediatrics and physiotherapy) that treated patients from within Ethiopia and surrounding countries, including Egypt, Sudan, Yemen, and Syria.^32^ The hospital had around 100 inpatient beds by the early 1960s and could treat up to 150 outpatients per day. The number of seconded Soviet specialists gradually increased from 30 in 1962 to 42 by 1979.^33^

In contrast with Ethiopia and Iran, the Soviet Red Cross began to contribute to Cambodian healthcare at a time of increasingly cordial relations between the two countries. In August 1960, the Khmer-Soviet Friendship Hospital opened in the Cambodian capital of Phnom Penh. The idea of a hospital was conceptualized in 1956, the year in which Cambodian-Soviet diplomatic relations were established and when Cambodian Prime Minister Norodom Sihanouk visited the USSR.^34^ The hospital was designed, constructed, and equipped by the Soviet government, and the Soviet Red Cross was responsible for providing medications and medical personnel. Initially, the Soviet Red Cross sent 25 highly qualified specialists to Phnom Penh, including doctors and surgeons with expertise in oncology, neurology, cardiology, otorhinolaryngology, anaesthesiology, dermato-venerology, gynaecology, ophthalmology, and paediatric medicine.^35^ At the time of its opening, the hospital was the largest in Southeast Asia and well regarded for expertise in radiology, radiotherapy, pulmonology, and surgery.^36^ The Soviet-built hospital was just one of many grants and projects ‘gifted’ to Cambodia from diverse sources in the context of the Cold War, but the sheer scale of the hospital project gave the USSR enormous influence over the Cambodian medical field.^37^ By the early 1960s, the hospital had 500 inpatient beds and an outpatient clinic with the capacity to accommodate 500 appointments per day.^38^

Overseas hospitals brought a range of foreign policy benefits to the Soviet government. Through equipping and maintaining medical institutions abroad, the Soviet Red Cross contributed to the development of the USSR's reputation as a powerful ally of national leaders in the Global South, as well as the promotion of Soviet medicine as an alternative to Western medical aid. While the Soviet government recognized these benefits, they were unwilling to match rhetoric about the superiority of Soviet medicine and the USSR's leading role in global health with appropriate funding. This forced the Soviet Red Cross to adopt financial models that were at odds with the core tenets of Soviet medicine and that prevented the delivery of the socialized healthcare that was offered within the USSR.

The fate of Soviet Red Cross overseas hospitals was dependent on the Soviet government's foreign policy priorities and the financial constraints imposed by the CPSU. In 1957, when Moscow–Tehran relations were particularly poor, a member of the Soviet Red Cross's Executive Committee bemoaned the fact that the Tehran hospital was housed in dilapidated buildings that lacked running water and central heating.^39^ The hospital building was upgraded in the next decade following a broader rapprochement in Iranian-Soviet relations.^40^ In the early 1960s, the Soviet Red Cross moved the hospital into a brand new six-storey building, which they leased and equipped using funds provided by the Soviet government.^41^ Similarly, in Phnom Penh the Soviet Red Cross managed to secure a significant amount of additional funding from the CPSU's Central Committee in 1969 for the maintenance of the Khmer-Soviet Friendship hospital. The decision to complete major building repairs on the hospital was coincidently taken at a time when Moscow sought to increase its influence within East Asia.^42^

Even though the Soviet Red Cross was able to secure the additional money required to renovate its overseas hospitals, the limited funding available restricted how far Soviet medical professionals could serve the local population. The Soviet side did not fund the treatment of patients, which meant that accessing Soviet healthcare was not free, unless the hospital received significant subsidies from the government of the recipient country. Instead, Soviet Red Cross hospitals tended to operate on the principle of ‘self-sufficiency’, wherein payments for treatment were supposed to fund the salaries of local staff and expenses incurred from the hospital's general operation. This stood in stark contrast to the USSR's healthcare system, within which healthcare was funded by the national budget and accessing treatment was (in theory) free for the patient.^43^ The Soviet Red Cross running paid-for healthcare facilities while espousing the benefits of the Semashko model of free healthcare encapsulates the complexity of the USSR's development practices in the Global South, which were characterized by their mixture of centralized planning and market systems.^44^ Development work was guided by pragmatism, local conditions, and the desire to generate economic growth, and the concept of ‘non-capitalist development’ was more important that strict adherence to Marxist-Leninist theory.^45^ As in the case of other East European development projects in the field of healthcare, socialist medicine had to adapt to the local context, whether in the case of everyday operations or funding models.^46^

The ‘self-sufficient’ funding model posed problems because it forced Soviet healthcare into a competitive environment. In a 1970 report on the Addis Ababa hospital, Soviet Red Cross leadership bemoaned the ‘unspoken struggle for patients’ between the city's 18 hospitals and noted that patients were quick to scrutinize Soviet medical personnel and assess whether their treatment represented value for money.^47^ This situation placed pressure upon the Soviet Red Cross because medical errors and staff popularity had a significant impact on the hospital's ability to function. For example, in 1967–8 when the repeated mistakes of two surgeons caused a number of preventable deaths in the surgical department, the chief surgeon at the rival Princess Tsehai Memorial Hospital penned a scathing critique and the Soviet Red Cross hospital witnessed a drop in patient numbers.^48^ Throughout the 1960s, numerous individuals who held the position of hospital director asked Soviet Red Cross leadership to more thoroughly vet specialists before sending them to work in Addis Ababa because the persistent issues with the quality of medical personnel were damaging the hospital's reputation.^49^

Soviet healthcare operated within a competitive environment in Cambodia too, despite the fact that the Khmer-Soviet Friendship hospital operated on a different financial model. Unlike the Soviet Red Cross hospitals in Tehran and Addis Ababa, the Phnom Penh hospital was managed and administered by local staff, rather than Soviet specialists. The Cambodian side was responsible for covering all costs with the exception of salaries for Soviet staff and the medications provided by the Soviet Red Cross.^50^ Nevertheless, the Khmer-Soviet Friendship hospital competed for patients with a number of other medical institutions that were funded by foreign organizations and governments. In an August 1967 report, a representative of the Soviet Embassy in Cambodia reported that the hospital was primarily used by the poor because treatment was mostly free or extremely cheap when compared to hospitals run by the state or funded by the French.^51^ However, offering free treatment had resulted in ‘serious financial difficulties’ because the Cambodian government did not provide the necessary funding. To rectify the situation, the Cambodian staff who managed the hospital proposed to build a number of new wards where wealthy Cambodian patients could be treated for a substantial fee. Rather than dismissing this suggestion on ideological grounds, the Soviet Embassy recognized the benefits of wealthy patients subsiding free treatment, but could not support the construction of a new building because they were not confident that the Cambodian state could provide the funds to maintain it.^52^ The Embassy representative noted that the Soviet side should retain control over the management of any hospitals that were ‘gifted’ to ‘underdeveloped’ countries in the future so that the correct financial models could be applied to ensure the institution's continued functioning.^53^

Even though treatment was free, the fact that the Khmer-Soviet hospital was one of many funded by foreign powers forced Soviet medicine into an environment infused with Cold War competition. During an August 1967 meeting with the head of the team of Soviet specialists, hospital director Kim Vien reported that Cambodian doctors who had trained in Paris and been awarded high-level qualifications (*professeur agrégé*) ‘tend to question the qualifications of medical personnel at the Khmer-Soviet Friendship Hospital’ and generally saw the hospital as inferior.^54^ To rectify the situation, Kim Vien proposed that highly skilled Cambodian doctors working at the Khmer-Soviet Friendship Hospital could be sent to the USSR for internships, after which they could receive a diploma with the equivalent high-level qualification. To ensure that these doctors were placed on the same standing as their colleagues who trained in France, he insisted that the Soviet title for doctor (*vrach*) ought not to appear on the diploma because this was interpreted as the lower status position of ‘health officer’ when it was translated into French. One month later in September, Kim Vien once again mobilized the language of Cold War rivalry to implore the Soviet Red Cross to send more specialists to work at the hospital. He regretted that he had recently dispatched one of the hospital's Cambodian doctors to France to train in anaesthesiology, but noted that this would not have happened if there had been a Soviet anaesthesiologist already in post.^55^

The Soviet Red Cross was upfront about the fact that their overseas hospitals operated on different financial models than Soviet institutions. An article celebrating the success of the Addis Ababa hospital published in *Sovetskii Krasnyi Krest* noted that while treatment was not free, fees were the lowest in the city and the institution was not a ‘commercial enterprise’ like hospitals in some Western countries.^56^ The article explained that fees were waived for patients who could not afford to pay, but did not provide further information on the prevalence of free treatment. From Soviet Red Cross reports, it is evident that free patients were a minority at the hospital. While members of the imperial family, state officials, and diplomats were regularly treated, just 71 of the 63,464 patients at the hospital received free treatment in 1969.^57^ The fact that the vast majority of patients paid for treatment suggests that one of the primary functions of the hospital was to showcase Soviet medicine, rather than to provide universal healthcare. This priority is evident in the numerous reports that were produced by the Soviet Red Cross leadership on other overseas hospitals, which tended not to include any information on free treatment and focused upon measures taken to popularize Soviet medicine and integratе Soviet specialists within the local scientific community.^58^ A significant number of patients at certain hospital departments were also Soviet citizens who had the privileges and political connections required to work abroad. For example, half of the 400 patients treated at the dental prosthetics department in the Soviet Red Cross hospital in Tehran in 1974 were seconded Soviet citizens employed at the Embassy, hospital management, or in other hospital departments.^59^ Although Soviet Red Cross hospitals did serve the local population, Soviet medicine was accessible only to those who could afford to pay or who had existing connections to the USSR.

The funding constraints placed upon Soviet humanitarian initiatives were a source of frustration for employees at overseas medical institutions, who often complainted that they did not have the resources to perform their roles effectively. In a July 1967 report for Soviet Red Cross leadership in Moscow, Mark Sorkin, the head of the team of Soviet specialists working at the Khmer-Soviet Friendship hospital, bemoaned the fact that they were ‘denied the most insignificant expenses, including office supplies and dictionaries’.^60^ He also noted that Soviet specialists were not provided with enough funding to cover their journeys to Cambodia. Because there were no direct flights from Moscow to Phnom Penh, Soviet specialists usually had to spend a couple of nights in Bangkok *en* route. They were allocated $5 per day spent in transit, but Sorkin pointed to recent cases of hospital doctors having to spend a minimum of $30 per day on accommodation and food in Bangkok.^61^ In Tehran throughout the 1970s, requests for new hospital equipment were frequently denied by the Soviet Red Cross Executive Committee in Moscow and local hospital staff complained about the fact that their wages were significantly lower than in other medical institutions in the Iranian capital.^62^

The CPSU's tight control over the scope and budget of Soviet Red Cross overseas work meant the organization could not take the initiative and quickly respond to the healthcare needs of local populations. In 1970, the Ethiopian Red Cross asked the Soviet Red Cross to provide free treatment for a small number of patients from Gambela region, around 700 km east of Addis Ababa.^63^ At that time, the Swedish Red Cross were working in the region on a multi-million-dollar project related to African trypanosomiasis prevention. The Ethiopian Red Cross informed the Soviets that they would only need to treat a maximum of two patients a month and that they would cover all transportation costs. Even still, Soviet Red Cross representatives in Addis Ababa could not readily offer support and had to send a formal request back to Moscow and await a decision. In the early 1980s, the Soviet Red Cross was called upon to step up their support to Ethiopia in the wake of devastating drought and famine.^64^ Throughout 1984–5, the Ethiopian government pursued a forced resettlement programme with the aim of moving millions of victims of famine and drought, which resulted in tens of thousands of excess deaths, ecological degradation, and devastating health and social consequences.^65^ The Ethiopian government called upon socialist countries to provide medical assistance to the resettlement programme, primarily through constructing and equipping new hospitals and clinics in remote regions that had been earmarked for resettlement. The Soviet Red Cross supported the establishment of field hospitals in a number of remote towns. One was opened in Asosa, a town 700 km east of Addis Ababa near the Sudanese border in 1984, and a mobile medical team were dispatched from Kyiv to work there. The Soviet Red Cross also sent surplus medical equipment from their Addis Ababa warehouse to hospitals in Debre Zeyit (now Bishoftu) and Nazret.^66^

Although the Soviet Red Cross was willing to provide temporary assistance, the organization was unable to commit to building and equipping new hospitals on a permanent basis. In a letter to Soviet Red Cross leadership, the director of the Addis Ababa noted that ‘it will be very difficult to charge for medical care’ and therefore, the issue of opening another hospital in Ethiopia ‘should be carefully studied’.^67^ This lack of commitment was connected to the fact that Soviet humanitarian work was intertwined with the foreign policy priorities of the Soviet government, whose enthusiasm assisting Ethiopia was rapidly waning by the mid-1980s because of their frustration with Mengistu's disastrous attempts to build socialism and his increasing demands for socialist bloc assistance.^68^ In contrast to the USSR, other countries from the socialist world were more willing to provide assistance. The GDR built, equipped, and staffed a hospital in Metema and Cuba sent dozens of doctors and other medical personnel.^69^ While Soviet Red Cross leadership were keen to present their organization as the leading humanitarian force in the socialist world, the CPSU were not willing to offer the funding required to cement this reputation.^70^

In addition to funding constraints, the Soviet Red Cross had to navigate numerous issues related to staff recruitment and retention. Overseas work was highly ideologically sensitive and representing the USSR abroad was a privilege afforded only to medical personnel who possessed the correct biography and credentials. Soviet staff working abroad had to send references to the Soviet Red Cross leadership in Moscow every ten months vouching for their behaviour and performance at work, and extending secondment beyond a year was a complex process that required the approval of the local Soviet embassy.^71^ Governments of recipient countries also had their own ideas about the kinds of Soviet specialists they wanted and used their visa regimes to achieve their own ends.

The Soviet Red Cross hospital in Tehran had persistent issues with staffing that resulted from decisions made both by the CPSU and the Iranian government. In 1956, 40 per cent of available positions in the hospital were not filled because the Soviet government kept recalling staff back to the USSR for ‘misbehaviour’ and organizing replacements was difficult due to the CPSU's lengthy process of vetting and approving candidates.^72^ This situation was enormously frustrating for Soviet Red Cross leadership, who frequently complained about the impossible task of assembling a pool of medical professionals who met the CPSU's requirements. To make matters worse, in 1958 the Iranian authorities stopped issuing entry visas, which meant that almost half of all positions at the hospital were vacant by the following year.^73^ In February 1964, Soviet Red Cross chairman Grigorii Miterev met with the Iranian Ambassador to the USSR in Moscow to discuss the visa issue. The Ambassador reminded Miterev that ‘modern Iran is not the Iran of the 1920s’ and that the country now had numerous qualified doctors and nurses, so was less dependent on Soviet specialists to deliver medical care.^74^ While the Ambassador confirmed that the Iranian government would gladly issue visas to Soviet specialists working in fields underrepresented by local specialists (such as neurology and neuropsychology), he insisted that there was no need for mid-level medical professionals to be sent from the USSR to Iran because ‘local personnel can be freely used’.^75^ The issue of securing Iranian visas remained a problem into the next decade and the hospital's director struggled to ensure that all departments were fully staffed throughout the 1970s.^76^

The Soviet Red Cross similarly struggled to send the agreed number of specialists to the Khmer-Soviet Friendship hospital because they did not have a reserve of medical professionals ready to be sent to Cambodia. To rectify this, in 1967 Soviet Red Cross leadership recommended ‘expanding the number of regions and republics from which we draw personnel’ and drew up plans to visit medical institutes in Alma-Ata, Tashkent, Baku, Yerevan, and Dushanbe to select ‘good specialists from local nationalities along with Russian comrades’.^77^ Although staff lists from Soviet Red Cross hospitals do not provide information on the ethnicity of Soviet specialists, from their surnames we can deduce that the majority of those working in at the Addis Ababa and Tehran hospitals were Russians and Ukrainians. In 1962, when building up a reserve of specialists for overseas secondment, the Soviet Red Cross recruited solely from cities in the Russian and Ukrainian republics, including Astrakhan, Leningrad, Ulyanovsk, Volgograd, Dnepropetrovsk, Kyiv, Kharkiv, and Zaporizhzhia.^78^

Given the fact that representing the USSR abroad was highly politically sensitive task, it is likely that seconded Soviet staff came from cities with the largest and most prestigious medical and research institutes. These were more often than not located in the Russian and Ukrainian republics because of the spatial inequalities that pervaded Soviet medicine. The fact the Soviet Red Cross leadership only looked to systematically recruit specialists from the Caucasus and Central Asia because reserves could not be drawn from their usual institutions is indicative of the Soviet government's limited investment in the so-called Soviet South as a model of development for the decolonizing world in the field of healthcare. From the late 1950s onwards, Soviet diplomatic initiatives instrumentalized Central Asia and the Caucasus to advance narratives about Moscow's commitment to anti-colonialism, championing of national cultures, tolerance of religion, and the achievements of Soviet development to an international audience.^79^ Leadership of the Soviet Red Cross only suggested expanding the pool of recruitment beyond the Russian and Ukrainian republics in the late 1960s, which indicates that showcasing the achievements of the Soviet South was not a major priority in this form of healthcare diplomacy.

Overseas hospitals were staffed by a mix of Soviet seconded personnel and locals, and while hospital directors generally believed that the two worked well together, there were persistent issues that affected relations between the two groups. Knowledge of local languages was not a prerequisite for overseas secondment, so most Soviet medical professionals working abroad could not communicate with local staff or patients without the mediation of their local colleagues. In a 1963 report, Soviet Red Cross leadership noted that local medical personnel in Addis Ababa and Tehran simultaneously worked as translators for Soviet staff, in addition to performing their clinical duties.^80^ This ignorance of local languages caused tension, and in the words of Soviet Red Cross leadership, ‘negatively affect[ed] the medical care of the population and complicate[d] contacts with the country's medical community’.^81^ In the case of Addis Ababa, Soviet Red Cross leadership recommended that seconded Soviet staff ought to study a foreign language while working at the hospital, and by 1970, two English language study groups had been established, as well as a Russian language group for Ethiopian staff. ^82^ Despite this, Soviet Red Cross leadership noted that Ethiopian medical personnel continued to act as translators for their Soviet colleagues alongside their other work duties in the years that followed.^83^ Perhaps in an attempt to rectify these issues, the Soviet Red Cross sent a team of five French translators to Phnom Penh to work alongside Soviet medical personnel at the Khmer-Soviet Friendship hospital. In addition to providing Russian-French translation during consultations between staff and patients, these translators held French language classes for Soviet medical personnel.^84^ Nevertheless, tensions between Soviet and local staff were ever-present. In the hospital's polyclinic, where Khmer and Soviet specialists worked side by side, the latter apparently found themselves ‘involuntary isolate[ed]’ because patients preferred to be treated by Khmer doctors.^85^

Other issues contributed to tensions between Soviet medical personnel and their Khmer colleagues and patients. The hospital's director, Kim Vien, had a good working relationship with seconded staff from the USSR, but the fact that the hospital was not Soviet managed posed a number of practical difficulties. Because they were not officially in charge, Soviet medical personnel did not have the authority to enforce standards around the storage of medications and their frequent requests to keep all medicines in an air conditioned room were ignored.^86^ Regardless of these issues, Soviet Red Cross leadership instructed Soviet medical personnel to ‘exercise maximum caution’ when making suggestions for how to improve hospital practices ‘so that [your] cooperation does not look like interference in the affairs of the administration’.^87^ Soviet staff were also deeply concerned by the impact of local traditions of care upon their ability to deliver treatment. In an October 1967 letter to Kim Vien, they requested that he reduce the ‘significant number of relatives and friends’ who remained at the bedside of hospital patients overnight, even on the tuberculosis ward.^88^ While Kim Vien was sympathetic to this request, he noted that the presence of relatives and friends was ‘characteristic of all hospitals in Cambodia’ and that ‘even the most drastic measures may prove useless’ because relatives would simply ‘find a way to enter the hospital’.^89^ Therefore, the Khmer-Soviet Hospital laid bare the incompatibility of Soviet medical practices and longstanding local traditions of care.

Staff composition at Soviet Red Cross overseas hospitals mirrored the specific dynamics of the Soviet healthcare system. In both Addis Ababa and Tehran, almost three quarters of Soviet staff were women.^90^ The gender imbalance at the hospital mirrored the highly feminized world of Soviet healthcare more generally, because women constituted over three quarters of the medical profession in the USSR.^91^ Despite this, increased feminization was accompanied by declining prestige for careers in medicine and healthcare, and just like in other aspects of the Soviet system, men tended to occupy the most prestigious and high-level positions.^92^ In the 1970s, women made up over 90 per cent of paediatricians, obstetricians, and gynaecologists working in the USSR, but only around 30 per cent of surgeons.^93^ At the Tehran hospital, women tended to work as deputy directors of departments, therapists, gynaecologists, radiologists, pharmacists, laboratory assistants, nurses, midwives, cashiers, secretaries, and accountants. In contrast, the hospital's director, director of the polyclinic, chief therapists, and the entire surgery department were all male.

The gendered hierarchies of Soviet medicine generated tension between seconded Soviet specialists in overseas hospitals. In October 1964, V. Pronin, the hospital's chief surgeon, wrote to the Soviet Red Cross Executive Committee to share his opinion on the replacement of his male colleague, surgeon N. Pavkin. Pronin shared the names of five men that he would recommend for the position, but was also clear on who he felt was unsuitable: ‘I kindly ask you not to send a woman. I resolutely believe that the phenomenon of the female surgeon is abnormal and unnatural’.^94^ The Soviet Red Cross Executive Committee flatly dismissed Pronin's request and sent a female surgeon to Tehran to replace Pavkin.^95^ Nevertheless, Pronin's outburst reveals how overseas hospitals reflected the contradictions of Soviet socialism more broadly, where rhetoric about gender equality brushed up against the gendered hierarchies of the medical profession.^96^

According to the Soviet Red Cross, their overseas hospitals were well regarded by the local population. In reports prepared for the CPSU, Soviet Red Cross leadership claimed that the reputation of their Tehran hospital meant that it was regularly used not only by city residents, but by individuals who travelled from Afghanistan, Saudi Arabia, and Pakistan specifically to receive treatment.^97^ The organization's magazine *Sovetskii Krasnyi Krest* regularly published testimonies from patients reflecting positively on their experiences at the hospital and singling out individual nurses, doctors, and surgeons who they believed had delivered exceptional care.^98^ Articles also included quotations from Iranian medical professionals praising the work of Soviet specialists and the import of advanced medical technologies from the USSR.^99^ Throughout the 1960s and 1970s, there were joint Iranian-Soviet medical conferences held in Tehran that facilitated collaborations between experts from both countries.^100^ In 1972, the Iranian Red Lion and Sun Society (the Iranian equivalent of the Red Cross) presented awards to hospital director Vasilii Goliakov and Soviet Red Cross chairwoman Nadezhda Troyan to recognize the hospital's significant contributions to public health in Iran.^101^

Although overseas hospitals received praise and recognition, depictions of these institutions in the Soviet Red Cross's magazine reveal the limitations of the anticolonial solidarities that were supposed to undergird these projects. The many articles profiling the Soviet doctors who worked in overseas locations were ‘underpinned by the cultural revival of fantasies of Western imperial power’ and presented them as intrepid explorers bringing civilization to racialized ‘others’.^102^ For example, in a 1963 group interview with the medical team about to begin their journey to Algeria, interviewees expressed pride in bringing the ‘achievements of Soviet medical science’ to Africa where local paramedics currently worked ‘in a primitive manner and did not use modern means of treatment’.^103^ Articles on overseas hospitals published in *Sovetskii Krasnyi Krest* were inflected by colonial discourses that contrasted socialist modernity with the ‘backwardness’ of postcolonial societies.^104^ In an interview with the magazine in 1970, Tehran hospital director Goliakov bemoaned the health impacts of fasting in Ramadan and detrimental effect of the ‘monotonous’ local cuisine upon patients with gastrointestinal conditions.^105^ When discussing a specific case of a patient who urgently required a blood transfusion, Goliakov contrasted the patient's ‘confused’ family members ‘who did not dare give blood’ with Soviet doctors, whose ‘high spirit of humanism’ caused them to offer their blood ‘without hesitation’. Similar discussions of doctors working abroad in Soviet Red Cross hospitals frequently emphasized that they were motivated by the high ideals of socialist humanitarianism, wherein the life of the collective was valued over individual comfort or preferences.

The selflessness of Soviet doctors working overseas was a common theme in articles published in *Sovetskii Krasnyi Krest,* but this framing elided the very tangible benefits that could accompany this kind of work. Because the Tehran hospital had a façade of neutrality, it was a useful site for gathering intelligence. This meant that some of the doctors were on the KGB and GRU payroll, according to former KGB officer Vladimir Kuzichkin who was stationed in Tehran in the 1970s and KGB-archivist-turned-defector Vasily Mitrokhin.^106^ Kuzichkin also claimed that the Tehran Soviet Red Cross hospital was a ‘goldmine’ for Petr Ashurko, the director at the time, and numerous high-profile doctors.^107^ Soviet doctors provided diagnostic services for Iranian specialists, after which they received half of the patients’ payment for treatment. Once they had obtained the money, the Soviet doctor could keep half for themselves and pay half to Ashurko, who made sure that this additional income was kept secret. Soviet doctors who participated in this scheme brought their income back to the USSR by befriending Soviet diplomats working in Tehran, who were exempt from customs inspections at both sides of the border because of their diplomatic passports.^108^ This practice appalled the majority of Soviet and Iranian employees at the hospital, but their complaints were ignored by Soviet officials in both Tehran and Moscow.^109^

Soviet Red Cross depictions of the Addis Ababa hospital followed similar trends to that of Tehran, where the ‘backwardness’ of the local population was presented in opposition to the ‘modernity’ of Soviet medical professionals. However, in the case of Addis Ababa, the theme of backwardness was employed both in relation to local customs and the development of socialism in the country more broadly. For example, a 1984 article in *Sovetskii Krasnyi Krest* that discussed a father's refusal to consent to the treatment of his son Samson likened Ethiopian society to that of the Soviet Union in the 1920s. When detailing the ‘diplomatic and oratory skills’ used by the doctors to change the father's mind, the author noted that ‘at such moments, one involuntarily recalls the experience of our doctors working in remote areas of the country during the early years of Soviet power, where they had to fight not only physical enemies, but also religious prejudices’.^110^ Samson was eventually treated at the Soviet Red Cross hospital, but his father later decided to discharge him and take him to a hospital run by Western specialists. Reflecting upon this, the article's author noted that the ‘class struggle [in Ethiopia] had not yet subsided’, which meant that the Soviet Red Cross hospital had become an ‘object of ideological sabotage’ by ‘enemies of the revolution’. The actions of Samson's father were presented as emblematic of the fact that Ethiopia was only in the early stages of building socialism and therefore, needed to learn from the Soviet Union. Here, Ethiopia was presented as a ‘younger socialist brother’ to whom Soviet scholars and policymakers could impart knowledge.^111^

Soviet Red Cross leadership presented the work of their seconded specialists as a selfless and powerful expression of solidarity with peoples in the decolonizing world. While certain medical personnel were undoubtedly motivated by their desire to deliver healthcare to people in need, this framing elided the benefits that came with overseas work. Publications produced by Soviet Red Cross leadership were rife with colonial discourses that contrasted the modernity of socialist medicine with the supposed backwardness of postcolonial societies and positioned Soviet personnel as intellectually superior to the populations that they served. The meshing of discourses of solidarity with a Soviet superiority complex is indicative of the interplay between national pride and internationalist values that underwrote Soviet postwar internationalism.^112^

The success of Soviet Red Cross hospital projects was not only affected by the CPSU's foreign policy priorities, the availability of funding and personnel, and narratives of Soviet civilizational superiority. The principal factor that decided the fate of these institutions was local politics, which was subject to abrupt and radical transformation within the volatile context of the Cold War. Throughout the 1970s, regime change occurred in Iran, Cambodia, and Ethiopia, and each new government radically reconfigured their relationship with the Soviet Union.

In Tehran, the fate of the Soviet Red Cross hospital changed rapidly after the Islamic revolution. The USSR's invasion of Afghanistan in December 1979 generated an upsurge in anti-Soviet sentiment across broad segments of Iranian society.^113^ The Soviet Red Cross continued to attempt to foster good relations with Ayatollah Khomeini's government through delivering healthcare assistance and humanitarian aid. Immediately after the revolution in February 1979, the Soviet Red Cross reportedly delivered 500litres of preserved blood and medications to Iran.^114^ In 1980, the organization delivered medications, baby formula, and blankets after severe floods displaced thousands of civilians in the southern province of Khuzestan.^115^ However, just like with other overtures from the Soviet side, the reception of the Iranian authorities was lukewarm. Tehran hospital director Dmitrii Sirak complained that the Iranian Red Crescent followed their expressions of gratitude with a reminder that ‘the Iranian people can cope with any difficulties themselves’.^116^ Sirak was also offended by the ‘simple’ ceremony organized to hand over the aid shipments and the fact that the head of the Iranian Red Crescent had chosen not to attend.

The hospital in Tehran continued its operations for the first few years under the new regime, even in the face of mounting pressure from the Iranian authorities. In April 1981, the President of the Iranian Red Crescent inspected the hospital and made a number of hostile and critical remarks. He demanded that the Soviet government provide more funding; insisted that all medical reports be written in English and Farsi, rather than Russian; and even asked why an Iranian hospital had not yet been established in Moscow.^117^ In October, the Iranian Ministry of Labour ordered the hospital to establish an Islamic Council to oversee staff behaviour, dress, and political attitudes ‘regardless of the wishes and consent of the administration’.^118^ In mid-December, Soviet staff at the hospital had been instructed to obtain new work permits within 24 hours or face huge fines and even immediate deportation.^119^ The Iranian Ministry of Finance insisted that the hospital had been paying the incorrect amount of tax and ordered that all financial documentation be surrendered to the government.^120^ The Ministry of Foreign Affairs also stopped issuing visas to Soviet specialists and the Ministry of Health forbade the importation of medications from the USSR.^121^

With pressure mounting from all sides of the Iranian bureaucracy, the Soviet Red Cross hospital in Tehran soon became untenable. In late December 1981, Iranian authorities informed the hospital director that their tax bill could be written off if the Soviet Red Cross agreed that all hospital property would be transferred to ‘the Iranian people’ free of charge in the event of the hospital's closure.^122^ Although it is difficult to verify whether this actually happened using archival evidence, we can assume that the Soviet Red Cross withdrew from the hospital at some point in 1982 because this is when the archival trail stops.^123^ It is highly likely that all Soviet specialists working at the hospital were recalled to the USSR at some point in 1982–3 following the defection of KGB officer Vladimir Kuzichkin and the expulsion of 18 Soviet diplomats who had been accused of espionage.^124^ In 1983, Ayatollah Khomeini's government unleashed a brutal crackdown on Iranian communists, which culminated in mass arrests and the disbandment of *Tudeh*.^125^

In Cambodia, the Khmer-Soviet Friendship hospital also fell victim to rapid regime change. After the 1970 coup against Prince Norodom Sihanouk, Grigorii Miterev, chairman of the Soviet Red Cross, wrote to the Central Committee of the CPSU stating his intention to recall all Soviet hospital employees because the Cambodian authorities were mainly using the hospital to treat military personnel and excluding Soviet medical specialists from this work.^126^ The hospital's director, Kim Vien, implored Soviet staff to stay and asked for the Soviet Red Cross to urgently send both medications and military surgeons.^127^ Following this, Soviet Red Cross leadership secured permission from the USSR's Council of Ministers to continue providing shipments of medication and equipment and to extend the stay of the seven Soviet hospital employees, who remained there until August 1972.^128^

It is difficult to ascertain exactly when Soviet medical personnel were recalled to the USSR, but this would have certainly happened at some point before the severing of Cambodia-USSR relations in 1973.^129^ Following the fall of Phnom Penh to the Khmer Rouge in April 1975, a widespread programme of mass violence, genocide, and the decimation of all branches of societal infrastructure began. As with all aspects of social, economic, and cultural life, the Khmer Rouge vowed to wipe the slate clean with healthcare. From 1975, hospitals and clinics were evacuated, medical equipment and medications were destroyed, and the vast majority of medical personnel who were educated in Western-style medicine were killed.^130^ The Khmer-Soviet Friendship Hospital was renamed the 17 April Hospital and functioned only for high-ranking members of the Khmer Rouge leadership.^131^

Soviet healthcare cooperation resumed following the fall of the Khmer Rouge and the establishment of the Vietnam-backed and supervised People's Republic of Kampuchea in January 1979. In 1980, the Soviet Red Cross sent a team of medical professionals to Phnom Penh under the auspices of the International Committee of the Red Cross, along with shipments of vaccinations, medical equipment, and medications.^132^ Soviet medical teams were assigned to a hospital 12 km south of Phnom Penh where they worked alongside medical teams from Cuba, the GDR, and Vietnam.^133^ The Khmer-Soviet Friendship Hospital tentatively reopened under its original name in 1982, but it did not reach full operational capacity until 1987.^134^ By this point, Soviet foreign policy in the Global South had shifted decisively towards reducing expenditure and cutting assistance in numerous fields, so the hospital could no longer rely on the ‘friendship’ of the Soviet government and the Soviet Red Cross that had been so forthcoming a few decades earlier.^135^ Nevertheless, the Khmer-Soviet Friendship Hospital outlasted the Soviet Union and continues to operate in the present day under its original name.^136^

In contrast to the hospitals in Tehran and Phnom Penh, the Soviet Red Cross hospital in Addis Ababa continued its operations uninterrupted up until, and even beyond, the Soviet collapse. In 1974, the ousting of Haile Selassie and the new Provisional Military Administrative Council's (Derg) drift towards Marxism-Leninism throughout the following year recalibrated Moscow-Addis Ababa relations. After a cautious beginning, Soviet support evolved from largely rhetorical to providing military assistance following Somalia's invasion of the Ogaden in July 1977. Throughout the late 1970s and early 1980s, Soviet military, technical, and economic aid flowed to Ethiopia as the Mengistu government decisively orientated the country towards the socialist world.^137^

The Soviet Red Cross hospital benefitted from closer Moscow-Addis Ababa relations. In 1977 and 1978, new departments were constructed, major renovations were completed on numerous existing buildings, new medical equipment was purchased, additional staff members were hired, and staff living quarters were upgraded.^138^ The Soviet government awarded the hospital the Order of the Friendship of the Peoples in 1978 to recognize the institution's contributions to Ethiopian public health and the ‘development and strengthening of friendly Soviet–Ethiopian relations’.^139^ In summer 1979, the Ethiopian Ministry of Health turned to representatives of state socialist countries (including the Soviet Red Cross) to ask for help in breaking the monopoly that foreign religious and charitable missions had upon Ethiopian healthcare.^140^ The director of the Soviet hospital took this request seriously and submitted fully costed plans to the Soviet Red Cross leadership in Moscow for new hospital branches in Gimbi and Aira, but these were never approved.^141^ Instead, Soviet Red Cross leadership continued to focus all their attention on their Addis Ababa hospital and opted for cheaper alternatives to support the development of Ethiopian healthcare, including providing first aid training for Ethiopian Red Cross volunteers and sending Soviet specialists to work at the free clinics run by the Ethiopian Red Cross throughout the 1980s.^142^ After the Soviet collapse, the institution was inherited by the Russian Red Cross and renamed the Russian Red Cross hospital. Today, it remains open and is financially supported by Vladimir Putin's administration.^143^

A core part of the Soviet Red Cross's international humanitarian programme was the delivery of Soviet healthcare overseas. As the trajectories of these three hospitals have indicated, the organization's ability to deliver healthcare abroad was shaped by the foreign policy priorities of, and the funding constraints imposed by, the CPSU. These limits generated personnel issues within hospitals and pushed the Soviet Red Cross to adopt funding practices that were incompatible with the core principles of Soviet medicine, including charging patients for treatment. When it came to finding Soviet specialists who were suitable for secondment, the Soviet Red Cross struggled to find candidates that would fit both the role profile and possess the political biography required to engage in international work. Leadership of the Soviet Red Cross tended to recruit specialists from republics with the largest and most prestigious medical and research institutes (Russia and Ukraine) and showed limited interest in presenting the Soviet South as a model for development in the field of healthcare. The CPSU's involvement in the work of the Soviet Red Cross meant that their humanitarian action was largely centred upon adapting Soviet healthcare practices to capitalist environments, all while showcasing the advancements of Soviet medicine.

In Soviet Red Cross hospitals, ideas about the unique ‘selflessness’ of Soviet humanitarians and the superiority of the Soviet system existed uneasily alongside the existence of paid healthcare. When the Soviet Red Cross attempted to smooth over the contradictions between healthcare policy at home and abroad, they emphasized the comparative affordability of treatment at their institutions and focused instead on the superiority of Soviet medicine and healthcare. This framing reflected broader hierarchies of Soviet internationalism, wherein the Soviet Union was placed in a dominant position as the chief arbiter of internationalist initiatives or projects on the global stage.^144^ The Soviet Red Cross's coverage of their overseas hospitals tended to draw sharp binaries between ‘developed’ Soviet medicine and the ‘backwards’ practices of local populations, which in turn reflected dominant assumptions about Soviet civilizational superiority in relation to peoples of the Global South and demarcated the limits of anticolonial solidarity.

## Notes

1 ‘S missiei gumanizma’, *Sovetskii Krasnyi Krest* (*SKK*) 12 (1970), 10–11.

2 On the early medical missions of the Soviet Red Cross in the 1920s, see Pavel E. Ratmanov and Vsevolod Y. Bashkuev, ‘Foreign Representative Offices of the Soviet Red Cross in the 1920s and 1930s in the Context of the International Health Policy of the USSR’, *History of Medicine,* 7, 1 (2021), 41–50.

3 Severyan Dyakonov, ‘“Resilience, Perseverance, and Sense of Diplomacy”: The Soviet Red Cross in India, 1954–1963’, *European Journal for the History of Medicine and Health,* 82 (2025), 78.

4 On de-Stalinisation and medical cooperation, Paula A. Michaels, ‘Soviet Medical Internationalism and Destalinization, 1953–1958’, *Soviet and Post-Soviet Review,* 50 (2023), 40–63.

5 On the intellectual and practical contours of this early engagement in the Global South, see chapter two of Alessandro Iandolo, *Arrested Development: The Soviet Union in Ghana, Guinea, and Mali, 1955–1968* (Ithaca 2022).

6 Oscar Sanchez-Sibony, ‘The Cold War in the Margins of Capital: The Soviet Union's Introduction to the Decolonized World, 1955–1961’, in James Mark, Artemy M. Kalinovsky and Steffi Marung (eds) *Alternative Globalizations: Eastern Europe and the Postcolonial World* (Bloomington 2020), 62–3; Artemy M. Kalinovsky, *Laboratory of Socialist Development: Cold War Politics and Decolonization in Soviet Tajikistan* (Ithaca 2018), 8.

7 One notable exception is Paula A. Michaels and Stéphanie Panichelli-Batalla, ‘An Unbreakable Friendship: Soviet Medical Internationalism in Cuba in the 1960s’, *History Australia,* 21, 4 (2024), 642–60. The scholarship on socialist medical internationalism is vast, so a select number of examples include Iris Borowy, ‘Medicine, Economics, and Foreign Policy: East German Medical Academics in the Global South During the 1950s and 1960s’, in Philip E. Muehlenbeck and Natalia Telepneva (eds) *Warsaw Pact Intervention in the Third World: Aid and Influence in the Cold War* (London 2018), 173–96; Dora Vargha, ‘The Socialist World in Global Polio Eradication’, *Revue d’études comparatives Est-Ouest,* 1, 1 (2018), 71–94; Dora Vargha, ‘Technical Assistance and Socialist Internationalist Health: Hungary, the WHO, and the Korea War’, *History & Technology,* 36, 3–4 (2020), 400–17; Young-sun Hong, *Cold War Germany, the Third World, and the Global Humanitarian Regime* (Cambridge 2015); Bogdan C. Iacob, ‘Paradoxes of Socialist Solidarity: Romanian and Czechoslovak Medical Teams in North Korea and Vietnam (1951–1962)’, *Monde(s)*, 2, 20 (2021), 117–40; Dongxin Zou, ‘Economizing Socialist Aid: China's Failed Surgical Plant in Algeria, 1973–80’, *Technology and Culture*, 63, 3 (2022), 718–48; Dongxin Zou, ‘Socialist Medicine and Maoist Humanitarianism: Chinese Medical Missions to Algeria, 1963–1984’, PhD dissertation Columbia University (2019); Dora Tot, ‘Translating Humanitarian Aid into Technical Cooperation: Yugoslavia's Medical Assistance to the Algerian National Liberation Movement (1958–1965)’, *International History Review,* 46, 6 (2024), 769–84; Cheasty Anderson, ‘South-South Cooperation as a Cold War Tonic: Cuban Medical Diplomacy to Sandinista Nicaragua, 1979–1990’, in Anne-Emanuelle Birn and Raúl Necochea López (eds) *Peripheral Nerve: Health and Medicine in Cold War Latin America* (Duke University Press 2020), 241–66.

8 Bogdan C. Iacob, ‘Health’, in James Mark and Paul Betts (eds) *Socialism Goes Global: The Soviet Union and Eastern Europe in the Age of Decolonisation* (Oxford 2022), 274–77.

9 Although the Soviet government also provided healthcare assistance on a bilateral basis without the involvement of the Soviet Red Cross, the organisation tended to be involved in some capacity in the largest and most high profile overseas medical projects. For an example of bilateral assistance that did not involve the Soviet Red Cross, see Ruth J. Prince, ‘From Russia with Love: Medical Modernities, Development Dreams, and Cold War Legacies in Kenya, 1969 and 2015’, *Africa,* 90, 1 (2020), 51–76.

10 Siobhán Hearne, ‘Between Moscow and Geneva: The Soviet Red Cross and the International Red Cross Movement’, *Journal of Global History,* 20, 1 (2025), 65.

11 Anna Wilkinson, ‘The League of Red Cross Societies’ Development Programme, the 1964 South East Asian Forum, and the Silencing of Asia’, *Asian Studies Review,* 47, 4 (2023): 761–77.

12 Dyakonov, ‘“Resilience, Perseverance, and a Sense of Diplomacy”’, 75.

13 There are numerous examples of this framing in materials produced by the Soviet Red Cross, but to provide just one: L. Cherkasskaia, ‘Krasnyi krest mozhet sdelat’ mnogo’, *SKK,* 9 (1976), 26.

14 The Red Cross movement was supposed to be independent of political, economic, and religious influence and operate without the interference of any state authorities, but throughout its history there has been a habitual violation of this principle at the multilateral and national level, Wilkinson, ‘The League of Red Cross Societies’ Development Programme’; David P. Forsythe, *The Humanitarians: The International Committee of the Red Cross* (Cambridge 2005), 183–92; Neville Wylie, Melanie Oppenheimer, and James Crossland ‘The Red Cross Movement: Continuities, Changes, and Challenges’, in Neville Wylie, Melanie Oppenheimer, and James Crossland (eds) *The Red Cross Movement: Myths, Practices, Turning Points* (Manchester 2020), 6–7.

15 On the centrality of ideas of civilisational difference to the creation of the postwar humanitarian regime, see Hong, *Cold War Germany,* 13–48.

16 Anna Calori, Anne-Kristin Hartmetz, Bence Kocsev, and Jan Zofka, ‘Alternative Globalisation? Spaces of Economic Interaction between the “Socialist Camp” and the “Global South”’, in Anna Calori, Anne-Kristin Hartmetz, Bence Kocsev, James Mark, and Jan Zofka (eds) *Between East and South: Spaces of Interaction in the Globalizing Economy of the Cold War* (Berlin 2019), 16.

17 Bogdan C. Iacob, ‘The Hospital: Uncomfortable Proximities – Romania's “One Nation Hospital” in Gharyan, 1974–1985’, in Kristin Roth-Ey (ed.) *Socialist Internationalism and the Gritty Politics of the Particular: Second-Third World Spaces in the Cold War* (London 2023), 177–96; Iris Borowy, ‘Medical Aid, Repression, and International Relations: The East German Hospital at Metema’, *Journal of the History of Medicine and Allied Sciences*, 71, 1 (2015), 64–92.

18 On the ‘gifting’ of medicine, hospitals, and technology by socialist governments, see Iacob, ‘Health’, 278; Michaels and Panichelli-Batalla Ruth, ‘An Unbreakable Friendship’, 642–43; Prince, ‘From Russia with Love’.

19 For a comprehensive overview of this shifting relationship, see James Mark and Yakov Feygin, ‘The Soviet Union, Eastern Europe, and Alternative Visions of a Global Economy, 1950s–1980s’, in James Mark, Artemy M. Kalinovsky, and Steffi Marung (eds) *Alternative Globalisations: Eastern Europe and the Postcolonial World* (Bloomington 2020), 35–58.

20 Michaels and Panichelli-Batalla, ‘An Unbreakable Friendship’, 643.

21 Fernande S. Raine, ‘The Iranian Crisis of 1946 and the Origins of the Cold War’, in Melvyn P. Leffler and David S. Painter (eds) *Origins of the Cold War: An International History*, (New York 2005), 93–112.

22 Susan Jones, ‘Evgeny Pavlovsky's Disruptive Science in Iran, 1941–43’, conference presentation at the International Council for Central and Eastern European Studies World Congress, University College London, 23 July 2025.

23 Nodar Mossaki and Lana Ravandi-Fadai, ‘A Guarded Courtship: Soviet Cultural Diplomacy in Iran from the Late 1940s to the 1960s’, *Iranian Studies,* 51, 3 (2018), 428–32.

24 Mossaki and Ravandi-Fadai, ‘A Guarded Courtship’; Roham Alvandi, ‘Flirting with Neutrality: The Shah, Khrushchev, and the Failed 1959 Soviet-Iranian Negotiations’, *Iranian Studies,* 47, 3 (2014): 419–40.

25 Rossiiskii gosudarstvennyi arkhiv noveishei istorii (Russian State Archive of Contemporary History, RGANI), f. 5, op. 55, d. 40, l. 50.

26 RGANI, f. 5, op. 55, d. 40, l. 50; Gosudarstvennyi arkhiv Rossiiskoi Federatsii (State Archive of the Russian Federation, GARF), f. R9501, op. 16, d. 149, ll. 65–8.

27 GARF, f. R9501, op. 16, d. 149, l. 1.

28 From 1896 to 1906, the imperial Russian Red Cross had managed a hospital in the city as part of the Russian government's broader attempts to thwart British and Italian ambitions in Abyssinia. Rashed Cowdhury, ‘Russian Medical Diplomacy in Ethiopia, 1896–1913’, in Anna Winterbottom and Facil Tesfaye (eds) *Histories of Medicine and Healing in the Indian Ocean World*, vol 2. (New York 2015), 115–45.

29 Radoslav A. Yordanov, *The Soviet Union and the Horn of Africa During the Cold War: Between Ideology and Pragmatism* (Lanham 2016), 6–9.

30 Yordanov, *The Soviet Union and the Horn of Africa,* 13–5.

31 Yordanov, *The Soviet Union and the Horn of Africa,* 17.

32 RGANI, f. 5, op. 55, d. 40, l. 50.

33 RGANI, f. 5, op. 55, d. 40, l. 50; GARF, f. R9501, op. 16, d. 197, ll. 9–12.

34 Jenna Grant, ‘“Cambodian Pathology”: Imagining Modern Biomedicine in the Cambodian-Soviet Medical Journal,* Revue Médico-Chirurgicale de l'Hôpital de l'Amitié Khméro-Soviétique* (1961–1971)’, in Hans Pols, C. Michele Thompson, and John Harley Warner (eds) *Translating the Body: Medical Education in Southeast Asia* (Singapore 2017), 202.

35 RGANI, f. 5, op. 55, d. 40, l. 50.

36 Jenna Grant, ‘Friends, Partners, and Orphans: Relations That Make and Unmake a Hospital’, *Medicine Anthropology Theory,* 5, 2 (2018), 58–60.

37 Cambodia received technical and economic assistance from the US, Japan, Czechoslovakia, Poland, and Yugoslavia from 1955 onwards, Grant, ‘Cambodian Pathology’, 198–9.

38 RGANI, f. 5, op. 55, d. 40, l. 50.

39 RGANI, f. 5, op. 40, d. 47, l. 126.

40 For an overview, see chapter three of Michael Pye, ‘In the Belly of the Bear?: Soviet-Iranian Relations during the Reign of Mohammad Reza Pahlavi’, PhD thesis, University of St Andrews (2015).

41 GARF, f. R9501, op. 16, d. 52, l. 205.

42 RGANI, f. 5, op. 61, d. 627, ll. 55–8.

43 Although the Soviet healthcare system was free at the point of access for patients, the situation was more complicated in reality and patients made informal payments to medical staff for a wide variety of treatments and services, Susan Grant, *Soviet Nightingales: Care under Communism* (Ithaca 2022), 216.

44 See chapter 4 of Iandolo, *Arrested Development.*

45 For an overview of the evolution of the concept of ‘non-capitalist development’ in the Khrushchev era, see Dmitry Asinovskiy, ‘Rostislav Ulianovskii, the Tudeh Party of Iran and Soviet Attempts to Set Iran on a Non-Capitalist Path of Development (1979–83)’, *Cold War History,* 24, 1 (2024), 47–9.

46 Iacob, ‘The Hospital’, 193.

47 RGANI, f. 5, op. 62, d. 636, l. 136.

48 RGANI, f. 5, op. 62, d. 636, ll. 134–5. The Princess Tsehai Memorial Hospital was opened in 1951 following an extensive fundraising and public interest campaign led by Sylvia Pankhurst. Sioban Nelson, ‘Nursing Experts, Hygienic Modernity, and Nation Building: The Case of Nursing in Ethiopia in the Post-Colonial Era’, *Canadian Bulletin of Medical History,* 38, 1 (2021), 63–92.

49 Rahvusarhiiv (National Archives of Estonia, ERA) R-2032.2.216, lk. 6; RGANI, f. 5, op. 55, d. 40, ll. 55–6.

50 Anne Yvonne Guillou, ‘Les médecins au Cambodge. Entre élite sociale traditionnelle et groupe professionnel moderne sous influence étrangère’, PhD dissertation, Ecole des Hautes Etudes en Sciences Sociales (2001), 145.

51 GARF, f. R9501, op. 16, d. 80, l. 151.

52 GARF, f. R9501, op. 16, d. 80, l. 146.

53 GARF, f. R9501, op. 16, d. 80, ll. 144–5.

54 GARF, f. R9501, op. 16, d. 80, ll. 75–6.

55 GARF, f. R9501, op. 16, d. 80, l. 126.

56 ‘Gospital’ otkrytii dliia vsekh’, *SKK,* 11 (1975), 28.

57 RGANI f. 5, op. 62, d. 636, l. 134.

58 See, for example, a Soviet Red Cross report on the Tehran hospital from 1963, GARF, f. R9501, op. 16, d. 52, ll. 196–8.

59 GARF, f. R9501, op. 16, d. 149, l. 5.

60 GARF, f. R9501, op. 16, d. 80, l. 43.

61 GARF, f. R9501, op. 16, d. 80, l. 44.

62 GARF, f. R9501, op. 16, d. 52, l. 137; GARF, f. R9501, op. 16, d. 149, l. 6.

63 RGANI, f. 5, op. 62, d. 636, l. 139.

64 On the development of these disasters and the Derg's exacerbating role, see Edmond J. Keller, ‘Drought, War, and the Politics of Famine in Ethiopia and Eritrea’, *Journal of Modern African Studies,* 30, 4 (1992), 609–24.

65 This resettlement scheme was part of a broader programme adopted by the Derg after their assumption of power to address a wide range of political and economic issues, including insurgency, overpopulation, and natural and mad-made disaster. On the programme and its profound consequences, see Africa Watch Reports, *Evil Days: 30 Years of War and Famine in Ethiopia* (London 1991), 211–30; Getachew Woldemeskel, ‘The Consequences of Resettlement in Ethiopia’, *African Affairs,* 88, 352 (1989), 359–74; Helmut Kloos, ‘Health Aspects of Resettlement in Ethiopia’, *Social Science and Medicine,* 30, 6 (1990), 643–56.

66 A. Sutormin, ‘Balcha – gospital’, *SKK,* 4 (1984), 27; GARF, f. 9501, op. 16, d. 269, ll. 15–6.

67 GARF, f. 9501, op. 16, d. 269, l. 41.

68 Yordanov, *The Soviet Union and the Horn of Africa*, 225.

69 Borowy, ‘Medical Aid, Repression, and International Relations’.

70 Hearne, ‘Between Moscow and Geneva’.

71 GARF, f. R9501, op. 16, d. 52, l. 100.

72 RGANI, f. 5, op. 47, d. 160, l. 21.

73 RGANI, f. 5, op. 47, d. 318, l. 158.

74 GARF, f. R9501, op. 16, d. 52, l. 15.

75 GARF, f. R9501, op. 16, d. 52, l. 16.

76 GARF, f. R9501, op. 16, d. 149, l. 76.

77 GARF, f. R9501, op. 16, d. 80, l. 135.

78 RGANI, f. 5, op. 55, d. 40, ll. 51–2.

79 Artemy M. Kalinovsky, ‘Writing the Soviet South into the History of the Cold War and Decolonisation’, in James Mark, Artemy M. Kalinovsky, and Steffi Marung (eds) *Eastern Europe and the Postcolonial World* (Bloomington 2020), 189–208; Albina Muratbekova, ‘Soviet Science Diplomacy: How Central Asia was Instrumentalised in Soviet Foreign Policy’, *Journal of Eurasian Studies,* 14, 1 (2023): 30–42.

80 RGANI, f. 5, op. 55, d. 40, l. 50.

81 ERA.R-2032.2.216, lk. 5.

82 RGANI, f. 5, op. 62, d. 636, ll. 135–6.

83 RGANI, f. 5, op. 62, d. 636, ll. 135–6.

84 GARF, f. R9501, op. 5, d. 862, l. 22.

85 GARF, f. R9501, op. 5, d. 862, l. 21.

86 GARF, f. R9501, op. 5, d. 862, l. 23.

87 GARF, f. R9501, op. 16, d. 80, l. 166.

88 GARF, f. R9501, op. 16, d. 80, l. 172.

89 GARF, f. R9501, op. 5, d. 862, l. 101.

90 GARF, f. R9501, op. 16, d. 52, ll. 23–4; GARF, f. R9501, op. 16, d. 217, l. 152; GARF, f. 9501, op. 16, d. 197, ll. 10–2.

91 Susan Grant, ‘Introduction’, in Susan Grant (ed.) *Russian and Soviet Health Care from an International Perspective: Comparing Professions, Practice and Gender, 1880–1960* (Cham 2017), 11.

92 Jeni Harden observes these trends within the female-dominated medical profession in Soviet and post-Soviet Russia, Jeni Harden, ‘“Mother Russia” at Work: Gender Divisions in the Medical Profession’, *The European Journal of Women's Studies,* 8, 2 (2001), 181–99.

93 Vincente Navarro, *Social Security and Medicine in the USSR: A Marxist Critique* (Lexington: Lexington Books, 1977), 78.

94 GARF, f. R9501, op. 16, d. 52, l. 83.

95 GARF, f. R9501, op. 16, d. 52, l. 94.

96 Iacob explores the Romanian hospital in Gharyan, Libya as a ‘highly gendered space that mirrored the politics and everyday mores of socialism in Romania’ where misogynistic attitudes and conversative ideas about sexuality existed alongside rhetoric about the ‘emancipation’ of women under Romanian socialism, Iacob, ‘The Hospital’, 188.

97 RGANI, f. 5, op. 50, d. 47, l. 127.

98 See for example, ‘Slova blagodarnosti’, *SKK,* 3 (1961), 25 and P. Ashurko, ‘Zdes’ dveri otkryty dliia vsekh’, *SKK,* 9 (1977), 11.

99 E. Azarashvili, ‘Zasluzhennoe priznanie’, *SKK,* 4 (1966), 23; E. Azarashvili, ‘Istoriia odnoi operatsii’, *SKK,* 9 (1969), 31–2.

100 ‘S missiei gumanizma’, 11; R. Brainina, ‘Chast’ dushi-patsientam’, *SKK,* 4 (1971), 9; GARF, f. R9501, op. 16, d. 149, l. 93.

101 RGANI, f. 5, op. 64, d. 656, l. 115; ‘Orden Krasnogo L’va i Solntsa – sovetskomu vrachu’, *SKK,* 3 (1972), 29.

102 James Mark, Anikó Imre, Bogdan C. Iacob, and Catherine Baker, ‘Introduction: Racial Disavowals – Historicising Whiteness in Central and Eastern Europe’, in James Mark, Anikó Imre, Bogdan C. Iacob, and Catherine Baker (eds) *Off White: Central and Eastern Europe and the Global History of Race* (Manchester 2024), 11.

103 ‘Kurs – Alzhir’, *SKK,* 2 (1963), 29.

104 Iacob, ‘Health’, 264, 279.

105 ‘S missiei gumanizma’, 10.

106 Vladimir Kuzichkin, *Inside the KGB: Myth and Reality,* translated by Thomas B. Beattie (London: Andre Deutsch, 1990), 160–6. GBR/0014/MITN 1/2: Iran, 1988, Typescript volumes compiled by Mitrokhin in Moscow, 1987–1992, The Papers of Vasily Mitrokhin, Churchill College Archives Centre, University of Cambridge. Prilozhenie 2: Spisok operativnikov, agentov, doverennikh. I am enormously grateful to Dmitry Asinovskiy for generously sharing this file with me, as well as for his guidance on KGB activity in Iran more generally.

107 Kuzichkin, *Inside the KGB,* 161.

108 Kuzichkin, *Inside the KGB,* 164.

109 Kuzichkin, *Inside the KGB,* 162.

110 ‘Balcha – gospital’, 27.

111 Diana L. Ohlbaum, ‘Ethiopia and the Construction of Soviet Identity, 1974–1991’, *Northeast African Studies,* 1, 1 (1994), 76–81.

112 Stefan B. Kirmse, ‘Internationalist Nation-Builders: Youth under Brezhnev in the Soviet South’, *Europe-Asia Studies,* 74, 7 (2022), 1254–77.

113 Asinovskiy, ‘“A Priest Does Not Consider the Toppling of the Shah as an Option”: The KGB and the Revolution in Iran’, *Iranian Studies**, * 55, 4 (2022), 944.

114 Iu. Ostal'skii, ‘K dobromu drugu i sosedu’, *SKK,* 5 (1980), 11.

115 R. Volkova and G. Kuz’micheva, ‘“Malysh” i “Zhar-ptitsa” letiat v Iran’, *SKK,* 5 (1980), 12–3.

116 GARF, f. R9501, op. 16, d. 217, l. 57. The Iranian Lion and Sun Society was renamed the Iranian Red Crescent in 1980.

117 GARF, f. R9501, op. 16, d. 217, ll. 120–8.

118 GARF, f. R9501, op. 16, d. 217, l. 24.

119 GARF, f. R9501, op. 16, d. 217, l. 11.

120 GARF, f. R9501, op. 16, d. 217, l. 41.

121 GARF, f. R9501, op. 16, d. 217, l. 42.

122 GARF, f. R9501, op. 16, d. 217, l. 30.

123 The fond of the Soviet Red Cross held in the State Archive of the Russian Federation (f. R9501) does not include any archival files on the Tehran hospital after 1981.

124 Artemy Kalinovsky, ‘The Soviet Union and the Iran-Iraq War’, in Nigel J. Asthon and Bryan R. Gibson (eds) *The Iran-Iraq War: New International Perspectives* (New York 2013), 237; Asinovskiy, ‘“A Priest Does Not Consider the Toppling of the Shah”’, 947.

125 Dmitry Asinovskiy, ‘The Soviet Union and the Iranian Revolution: How Experts, Intelligence Services, and Politicians of the Two Superpowers Missed the Birth of Islamic Fundamentalism’, *Russia in Global Affairs,* 16, 3 (2018), 195; Asinovskiy, ‘“A Priest Does Not Consider the Toppling of the Shah’’', 947.

126 RGANI, f. 5, op. 62, d. 636, ll. 105–6.

127 GARF, f. R9501, op. 5, d. 862, l. 127.

128 RGANI, f. 5, op. 62, d. 636, l. 179; GARF, f. R9501, op. 5, d. 862, l. 137.

129 The vast majority of staff at the Soviet Embassy in Phnom Penh were recalled to the USSR in October 1973, so it is reasonable to assume that Soviet medical personnel would have left before then. Sydney H. Schanberg, ‘All but 3 Soviet Aides Leave Cambodia’, *New York Times* (26 October 1973), 3.

130 James A. Tyner, ‘State Sovereignty, Bioethics, and Political Geographies: The Practice of Medicine under the Khmer Rouge’, *Environment and Planning D: Society and Space,* 30 (2021), 846–9.

131 Jenna Grant, ‘How to Rename a Hospital: Biomedical Technologies and New Combinations of Business and Charity in Cambodian Public Health’, *Anthropological Quarterly*, 90, 3 (2017), 610; Tyner, ‘State Sovereignty, Bioethics, and Political Geographies’, 851–2.

132 ‘Spetsreisy v Pnompen’’, *SKK,* 2 (1980), 5; ‘Bozrozhdaiushchaiasia iz pepla’, *SKK,* 6 (1980), 28–9. International Committee of the Red Cross Archives, B AG 121 218-015, Généralités. Telex from Geneva to Phnom Penh 21 August 1980; Telex from ICRC to Soviet Red Cross 16 August 1980.

133 International Federation of the Red Cross Archives, box Z000971, Correspondence H. Beer. Notes on debriefing of Mr Ivan Teterin, 11 February 1980. ‘Akun tiron – bol'shoe spasibo’, *SKK,* 2-3 (1981), 33.

134 Guillou, ‘Les médecins au Cambodge’, 271.

135 Svetlana Savranskaya, ‘Gorbachev and the Third World’, in Artemy Kalinovsky and Sergey Radchenko (eds) *The End of the Cold War and the Third World: New Perspectives on Regional Conflict* (New York 2011), 21–45.

136 Now it is financially supported by the Cambodian Ministry of Health and donations from international NGOs and foreign corporations. Grant, ‘How to Rename a Hospital’.

137 See chapters seven and eight of Yordanov, *The Soviet Union and the Horn of Africa;* Ohlbaum, ‘Ethiopia and the Construction of Soviet Identity’.

138 GARF, f. 9501, op. 16, d. 197, ll. 44–7.

139 S. Silishchev, ‘Orden druzhby narodov – bol’nitse Sovetskogo Krasnogo Kresta v Efiopii’, *SKK,* 5 (1978), 9.

140 GARF, f. 9501, op. 16, d. 197, l. 123.

141 GARF, f. 9501, op. 16, d. 197, ll. 217–46.

142 GARF, f. 9501, op. 16, d. 197, ll. 204–12.

143 In 2022, the Russian Red Cross signed an agreement with the Ethiopian Ministry of Health to continue the work of the hospital for the next 25 years: https://redcross.ru/news/rossiyskiy-krasnyy-krest-dogovorilsya-s-ministerstvom-zdravookhraneniya-efiopii-o-prodlenii-raboty-g/?sphrase_id=11675 (accessed 31 May 2024). The agreement was made possible through a grant authorised by Vladimir Putin for strengthening links between the Russian Red Cross and its international counterparts and promoting the use of the Russian language within the international Red Cross and Red Crescent movement: https://xn--80afcdbalict6afooklqi5o.xn--p1ai/public/application/item?id=44c03b7f-0bed-46a9-9fe5-8f34273aff2f (accessed 31 May 2024).

144 Kalinovsky, *Laboratory of Socialist Development,* 11.

